# Hedonism as a motive for information search: biased information-seeking leads to biased beliefs

**DOI:** 10.1038/s41598-023-29429-8

**Published:** 2023-02-06

**Authors:** Matthew Jiwa, Patrick S. Cooper, Trevor T-J. Chong, Stefan Bode

**Affiliations:** 1 University of Melbourne, School of Psychological Sciences, Melbourne, 3010 Australia; 2 Monash University, Turner Institute for Brain and Mental Health, Melbourne, 3800 Australia; 3 Department of Neurology, Alfred Health, Melbourne, 3004 Australia; 4 Department of Clinical Neurosciences, St Vincent’s Hospital, Melbourne, 3065 Australia

**Keywords:** Human behaviour, Computational science

## Abstract

Confirmation bias in information-search contributes to the formation of polarized echo-chambers of beliefs. However, the role of valence on information source selection remains poorly understood. In Experiment 1, participants won financial rewards depending on the outcomes of a set of lotteries. They were not shown these outcomes, but instead could choose to view a prediction of each lottery outcome made by one of two sources. Before choosing their favoured source, participants were first shown a series of example predictions made by each. The sources systematically varied in the accuracy and positivity (i.e., how often they predicted a win) of their predictions. Hierarchical Bayesian modeling indicated that both source accuracy and positivity impacted participants’ choices. Importantly, those that viewed more positively-biased information believed that they had won more often and had higher confidence in those beliefs. In Experiment 2, we directly assessed the effect of positivity on the perceived credibility of a source. In each trial, participants watched a single source making a series of predictions of lottery outcomes and rated the strength of their beliefs in each source. Interestingly, positively-biased sources were not seen as more credible. Together, these findings suggest that positively-biased information is sought partly due to the desirable emotional state it induces rather than having enhanced perceived credibility. Information sought on this basis nevertheless produced consequential biased beliefs about the world-state, highlighting a potentially key role for hedonic preferences in information selection and subsequent belief formation.

## Introduction

Despite the overwhelming availability of information, societal beliefs about a wide range of topics remain highly polarised. This is exemplified by beliefs about the likely outcome of the 2020 US presidential election in the weeks prior. According to Republican supporters, the incumbent Donald Trump had a 76% chance of victory, while Democratic supporters rated Joe Biden’s probability of winning at 71%^1^. Similar conflicting views are present in public perception of key issues, such as anthropogenic climate change^2^, which can lead to consequential failures to reach a consensus on how to address such pressing existential concerns. Given the epistemic threat posed by highly polarised beliefs^3^, it is important that we understand the mechanisms contributing to their development.

Importantly, individuals have been shown to selectively seek information from sources that overlap with their pre-existing beliefs^4–8^. This preference for consonant (rather than dissonant) information, termed confirmation bias (sometimes selection bias; congeniality bias), combines with the increasing diversity of available information sources to produce polarised belief structures^9–11^.

Recent accounts of confirmation bias argue that it may not be the product of bias, but instead of entirely rational Bayesian inference^6,12^. According to this explanation, individuals are inclined to sample more from sources that align with their prior beliefs, as information that aligns closely with one’s prior beliefs is seen as more believable than information that is more distal^13^. Indeed, the rational tendency to factor in one’s prior beliefs when evaluating the veracity of new information could account for divided beliefs on issues such as climate change^14^ and is sufficient to explain the formation of polarised echo-chambers of conflicting beliefs^15^.

A different, though not mutually exclusive, account of confirmation bias is that it arises from the pursuit (or avoidance) of information to maintain a desired emotional state, social identity, or belief state^7,8,16^. Theories of information valuation suggest that the pursuit of these differing goals may be attributable to the overarching principle of hedonic value—information is often sought or avoided due to the affective state it induces^17–20^. In support of this, it has been shown that people prefer early information about the outcome of lotteries when these have potential positive as opposed to negative consequences^21^, and when winning is more likely than losing^22^. As positive reward prediction errors have been shown to produce positive changes in subjective mood^23,24^, it is argued that this behaviour reflects the pursuit of hedonic goals^19,20^. Further, as concordant information is perceived as more pleasant than discordant information^4,8,16,25^, the preference for concordant information could be partially attributable to the positive affect it is expected to induce^7,8,26^.

The potential effects of the hedonic pursuit of desirable information on the beliefs we form are also poorly understood. Individuals with the most extreme beliefs have been shown to have the greatest confidence in their beliefs^27,28^. This may be driven by mental rigidity, which has been shown to co-occur both with extreme beliefs and the preference for congenial information^29,30^. However, given that positively-valenced information is believed more readily than negatively-valenced information^31–33^, the association between extreme beliefs and belief confidence may instead be a by-product of positively-biased information search. These data highlight the potential for confirmation bias to lead to extreme, polarized beliefs^11^. Those engaging in biased information selection may therefore exhibit higher confidence in the beliefs they form, although this is yet to be directly tested.

Past research into the motivations underlying confirmation bias have primarily focused on participants’ preferences for news articles based on real-world issues^5,6,9,12,34^. While these studies provide valuable insight into the magnitude, extent, and individual variability of confirmation bias, there are key limitations to this approach. Most notably, studies using this method are unable to provide a mechanistic account of information preferences, as they are unable to disentangle the contributions of rational Bayesian inference from the hedonic pursuit of desirable affective states. Second, these studies typically require participants to explicitly rate the perceived accuracy and credibility of the news articles they read—a process which can subsequently bias future responses^34^. Finally, participants vary widely with regards to their prior knowledge of the relevant news articles, which affects the extent to which presented news articles provide novel information. As a consequence, observed behaviour may generalize poorly to future information-seeking, which typically pertains to the unknown^19^. In the present study, we aimed to circumvent these issues using a novel methodology that involves no prior knowledge but maintains valenced outcomes. This approach allowed us to isolate the contribution of hedonism and to ensure behaviour is related to unknown information, increasing the validity of findings.

In summary, individuals are known to exhibit a confirmation bias, which subsequently leads to the formation of polarised belief structures. Two key properties of information have been proposed to lead to this bias: the proximity of information to one’s existing beliefs, and the affective content of that information. Importantly, however, the extent to which each contributes remains largely unknown, with some studies arguing that the hedonic pursuit of positive affect need not be present for confirmation bias to manifest^14^. In this study, we aimed to isolate the role of the hedonic value of information on source selection by manipulating its expected valence. In Experiment 1, we asked whether participants prefer more accurate sources of information relative to those that offer more positively-valenced information. According to the Bayesian rational argument, participants should choose to seek information only from the most accurate source, while the hedonic perspective suggests that participants will place value on sources with a higher likelihood of yielding positively-valenced information. This experiment also allowed us to ask how information preference impacts the beliefs we form and the confidence with which we hold those beliefs. In Experiment 2, we aimed to understand whether the preference for positively-biased information is attributable to the perception of positively-biased sources as more accurate than equivalent, unbiased sources.

## Results

### Experiment 1

**Figure 1:**
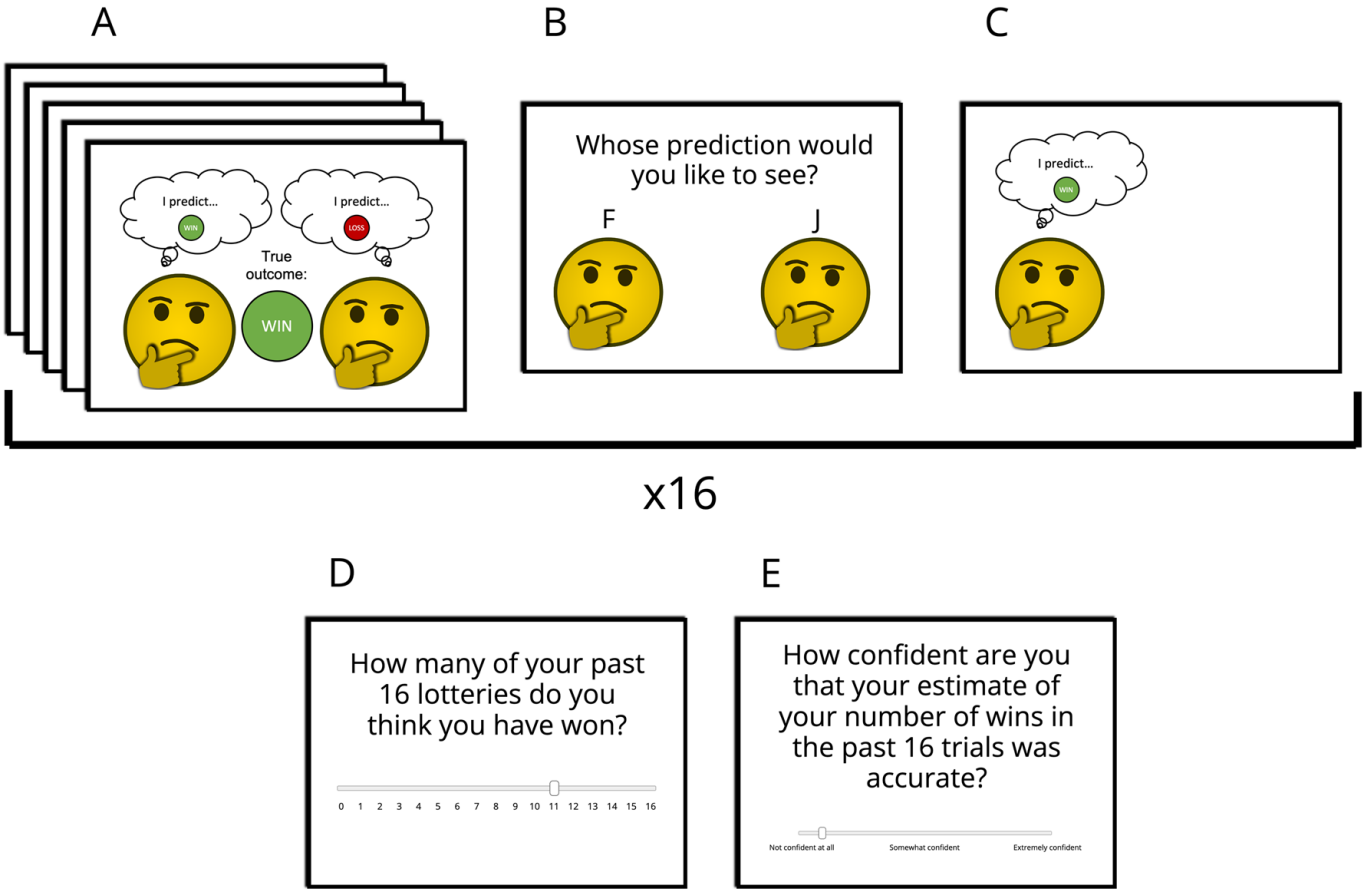
Experiment 1 paradigm. (**A**) Participants were first shown a series of five example predictions made by both psychics, with the true outcome of each example lottery shown. (**B**) They then selected which psychic’s prediction they would like to see for their own lottery. (**C**) Their chosen psychic then made a prediction for their lottery. The true outcome was not revealed. In each block, participants completed 16 trials. (**D**) After each block of trials, participants were asked to estimate the number of lotteries they had won within that block. (**E**) They also provided a confidence rating for their estimate on a continuous scale.

Participants completed a random lottery task in which, on each trial, they could either win (gain 20c) or lose (gain 0c). Each lottery consisted of a simple coin flip in which the green side indicated a win, and the red side indicted a loss (win probability of 50%). Before their lottery was played, they were shown two sources (termed “psychics” in the experiment), represented by standard emojis (Fig. 1), each of which made predictions about a series of five independent “example” lotteries (which were unrelated to the current trial). Psychics systematically varied in how accurate and how positive they were in their predictions for these five lotteries (i.e., they varied in the relative number of correct predictions, as well as in the ratio of “win” and “loss” predictions they made, respectively). All psychics made correct predictions on a minimum of three of the five example predictions. Following the observation of these example predictions, participants chose one of the psychics to make a prediction about their own lottery. Subsequently, they were shown the prediction of the lottery outcome by their chosen psychic, but they were not shown the respective outcomes (Fig. 1). Psychics that made more positive predictions in the example lotteries were more likely to make positive predictions for the participant’s lottery. Participants were told that all lottery earnings were added to their total and would be paid out at the end of the experiment.

Each participant completed four blocks of 16 trials. After each block, they provided an estimate of how many of the past 16 lotteries they believed they had won, and a confidence rating associated with how accurate they believed their estimate to be.

#### Preference for positively-biased predictors

**Figure 2:**
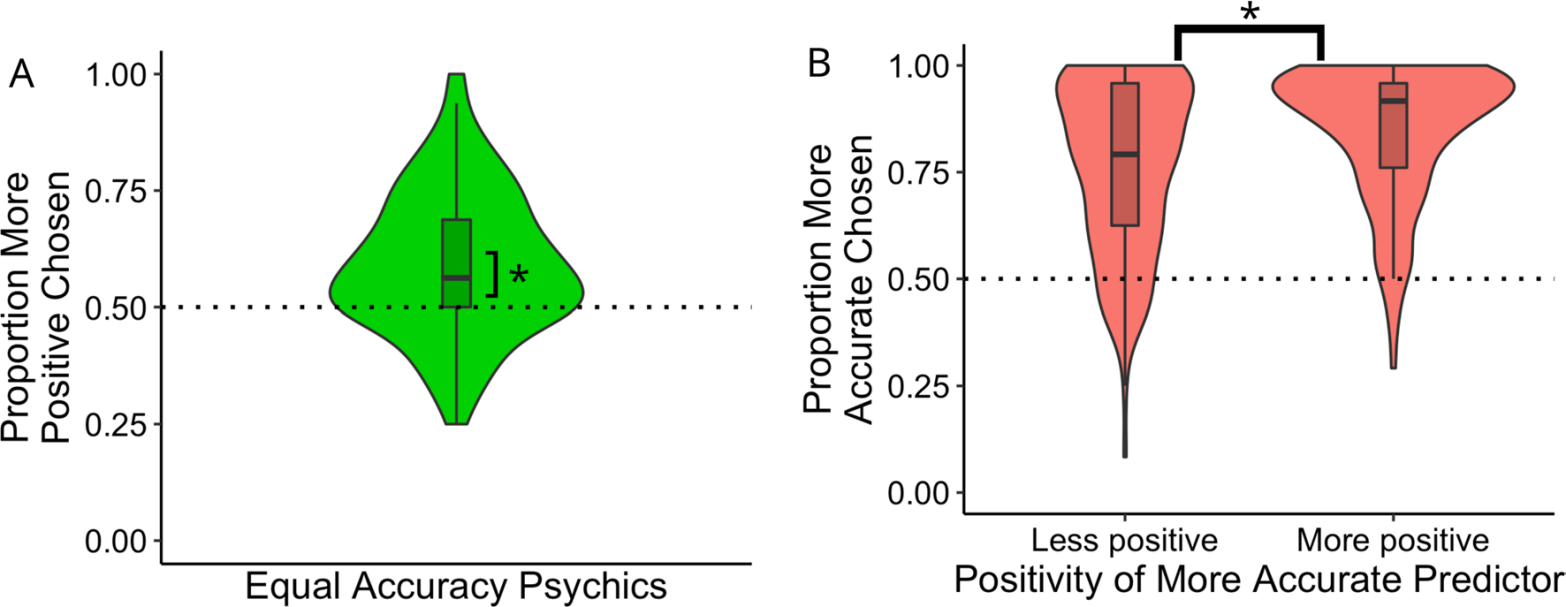
Behavioural results. (**A**) Trials in which the two psychics had equal accuracy in their example predictions. Plotted is the distribution of the mean proportion of trials in which participants chose to view the prediction of the more positive psychic. Asterisk denotes significant difference from chance level. (**B**) Trials in which the two psychics were not equally accurate, split by whether the more accurate psychic predicted fewer or more “win” outcomes than the less accurate psychic. Plotted are distributions of the mean proportion of trials in which the more accurate psychic was selected.

To assess whether participants showed a preference for more positive psychics (i.e., those with a tendency to predict more “win” outcomes than their counterpart), we assessed participants’ choice behaviour with respect to the example predictions made by each psychic. On trials in which the two psychics were equally accurate, participants showed an overall preference for the psychic that predicted more “win” outcomes in their example predictions, *t*(157) = 7.60, *p* < 0.001 (Fig. 2A). On trials in which the two psychics were not equally accurate, participants overwhelmingly preferred the more accurate psychic to the less accurate psychic, *t*(157) = 24.29, *p* < 0.001. However, preference for the more accurate psychic was higher on trials in which the more accurate psychic predicted a greater number of “win” outcomes than the less accurate psychic than on trials in which the more accurate psychic predicted fewer “win” outcomes than the less accurate psychic, *t*(157) = 6.40, *p* < 0.001 (Fig. 2B).

#### Modelling of positivity-preference

To gain a more detailed perspective of participants’ preferences, we fit a series of computational models to individual choice behaviour (see “Methods” for details). The first model assumed that the positivity of psychics’ predictions would have no effect on choice behaviour. In this *Accuracy Only Model*, participants’ choices were dictated only by how accurate the psychics were in their example predictions. In the second model (*Positivity Only Model*), participants’ choice behaviour was dictated only by the number of “win” outcomes the psychics predicted in their example predictions. The third model (*Accuracy & Positivity Model*) operationalised the hypothesis that both the accuracy and positivity of psychics’ example predictions would drive choice behaviour. Finally, in the *Accuracy & Positivity Tiebreak Model*, participants’ choices depended only on how accurate the psychics were, but, when the two psychics were equally accurate, the number of “win” predictions made was used as a tiebreak criterion.

Model fit was assessed using k-fold truncated importance sampling leave-one-out cross-validation (TIS-LOO-CV)^35^. This approach implicitly penalises model complexity, as more complex models will overfit to the training data, reducing their accuracy on the test set^36^. To optimise model predictions for extrapolation to new participants, the hierarchical structure of the data was incorporated into the construction of folds for cross-validation such that each iteration excluded the data of a single participant^37,38^. Participant-wise expected log posterior density (elpd) was estimated and summed across participants to provide an overall measure of model fit. Bayesian stacking was used to assess which models contributed to the optimal predictive performance across a weighted combination of all models^39^ (see “Methods” for details). We also report the Watanabe–Akaike Information Criterion^40,41^ to demonstrate that a statistic with an explicit penalty for complexity also produces the same findings. Results suggested that the *Accuracy & Positivity Model* produced the most accurate predictions and contributed most to the optimal weighted model (Table 1). Model recovery analyses demonstrated that the true generative model was identifiable with high accuracy (see Supplementary Tables A1, A2, and A3). Additionally, parameter recovery analysis for the best-fitting model indicated that all three of the model parameters were accurately recoverable (all p-values < 0.001; see Supplementary Table A4). Posterior predictive checks show an excellent fit of the *Accuracy & Positivity Model* to the data (see Supplementary Fig. A1). Final parameter estimates for the *Accuracy & Positivity Model* are shown in Supplementary Fig. A2.

Next, we tested our model predictions at the level of individual participants. Despite providing the best overall predictions of the data, the *Accuracy & Positivity Model* did not provide the best individual predictions for all participants. The *Accuracy Only Model* provided the best predictions for 46.2% of participants, and the *Accuracy & Positivity Model* for 31.0% of participants. Of the remainder, 12.0% of participants’ behaviour was best characterised by the *Accuracy & Positivity Tiebreak Model*, while the 10.8% were best predicted by the *Positivity Only Model* (see Table 1). Overall, these data demonstrate the sizeable individual differences in choice behaviour, as have been observed in previous studies in this area^42,43^.

**Table 1:** Experiment 1 model fit statistics. Estimated expected log-posterior density (elpd), Watanabe–Akaike Information Criterion (WAIC), Bayesian stacking weights, and the proportion of participants whose data was best predicted by each model. Larger positive values of elpd and smaller values of WAIC indicate better predictive performance. The difference from the best fitting model is represented as $\Delta {\hat{elpd}}$.

Model	${\hat{elpd}}$ (S.E.M)	$\Delta {\hat{elpd}}$ (S.E.M)	WAIC (S.E.M)	Bayesian weight	Prop. best predictor
Accuracy & Positivity Model	− 4813 (133.1)	–	9584.21 (104.22)	0.910	0.310
Accuracy Only Model	− 4973 (135.7)	− 161 (44.5)	9921.54 (100.67)	< 0.001	0.462
Positivity Only Model	− 6895 (37.5)	− 2083 (136.3)	13,760.70 (29.86)	< 0.001	0.108
Accuracy & Positivity Tiebreak Model	− 4919 (134.8)	− 106 (33.8)	9792.30 (102.60)	0.090	0.120

Bayesian stacking weights correspond to the weights assigned to the predictions of each model that maximise the log predictive density of future data.

#### Biased information-seeking and biased beliefs

Within our experimental design, positively-biased psychics had an increased likelihood of yielding positive predictions on participants’ lotteries. As a consequence, participants’ positivity-preference parameter (produced from fitting the *Accuracy & Positivity Model*) was strongly correlated with the number of wins participants’ chosen psychics predicted on the participants’ lotteries (*r* = 0.56, *p* < 0.001). To assess whether these biased predictions influenced the beliefs participants formed about the frequency with which they had won, we constructed a mixed-effects regression model. Within this model, we predicted the number of wins a participant estimated they had achieved within each block from the number of win outcomes their chosen psychics predicted on the participant’s lotteries in that block, combined with a participant-level intercept term (model specification: *winEstimates* $\sim$ *shownWins + (1 | ID)*). The model was estimated using standardised parameters in the lme4 R package^44^. Model output revealed a significant, positive effect of the number of win predictions the participant had been shown on the number of wins they estimated they had achieved, $\beta$ = 0.225, *SE* = 0.031, 95% CI = [0.165, 0.285]. This suggests that psychics’ predictions influenced participants’ beliefs about their lottery outcomes, with participants exposed to a greater number of positive predictions believing they had won more often.

#### Biased beliefs and confidence

It is plausible, however, that participants who sought positively-biased information were conscious of the bias in the information they received, and therefore adjusted the confidence with which they held beliefs about the number of wins they had achieved. To assess this, we examined the relationships between participants confidence ratings and their choice behaviour. In addition to the association between the positivity-preference parameter and the positivity of psychics’ predictions, there was a strong correlation between participants’ accuracy-preference parameter and the mean accuracy of their chosen psychics’ example predictions (*r* = 0.96, *p* < 0.001). To understand how both the accuracy of psychics’ example predictions and the valence of their predictions on the participants’ lotteries affected the strength with which participants believed the psychics, we constructed an additional mixed-effects model. Within this model, we predicted participants’ confidence ratings from the mean accuracy of their chosen psychics’ example predictions within a given block and the number of win outcomes their chosen psychics predicted on the participant’s lotteries in that block. We also included an interaction term between these two factors, and a participant-level intercept term (model specification: *confRatings* $\sim$ *1 + shownWins* $\times$ *meanAcc + (1 |ID)*) Model output revealed a significant main effect of the number of win predictions the participants chosen psychics had yielded, $\beta$ = 0.105, *SE* = 0.028, 95% CI = [0.050, 0.161] but not of the mean accuracy of the chosen psychics, $\beta$ = 0.060, *SE* = 0.039, 95% CI = [− 0.017, 0.136] nor the interaction between the two, $\beta$ = − 0.049, *SE* = 0.026, 95% CI = [− 0.100, 0.002]. These results suggest that the valence of participants’ chosen psychics’ predictions on the participants’ lotteries, but not the accuracy of their example predictions predicted higher confidence in participants’ estimates of their frequency of wins.

In summary, the results of Experiment 1 showed that the majority of participants engaged in positively-biased information search. Receiving positively-biased information was positively associated with the belief that they had won with a higher frequency, and with the confidence with which these beliefs were held.

### Experiment 2

One explanation for the results in Experiment 1 is that participants attend to or weight positive predictions differently to negative predictions. For example, when evaluating the credibility of a source, correct predictions of positive outcomes may be weighted more heavily than correct predictions of negative outcomes, leading to the perception of positively-biased predictors as more credible^45^. Alternatively, participants could be sampling from positively-biased information sources to achieve hedonic goals (i.e., simply because it makes them feel better, but not because they believe it is more credible). While this alone would not explain the increases in confidence for positively-biased information seekers, these differences could be due to the tendency for individuals to believe positive information more readily than negative information. This could be explained by asymmetrical belief updating (larger belief updates following positive relative to negative information)^31–33^, or to optimism bias (in which positive outcomes are expected and therefore more plausible)^46,47^.

**Figure 3:**
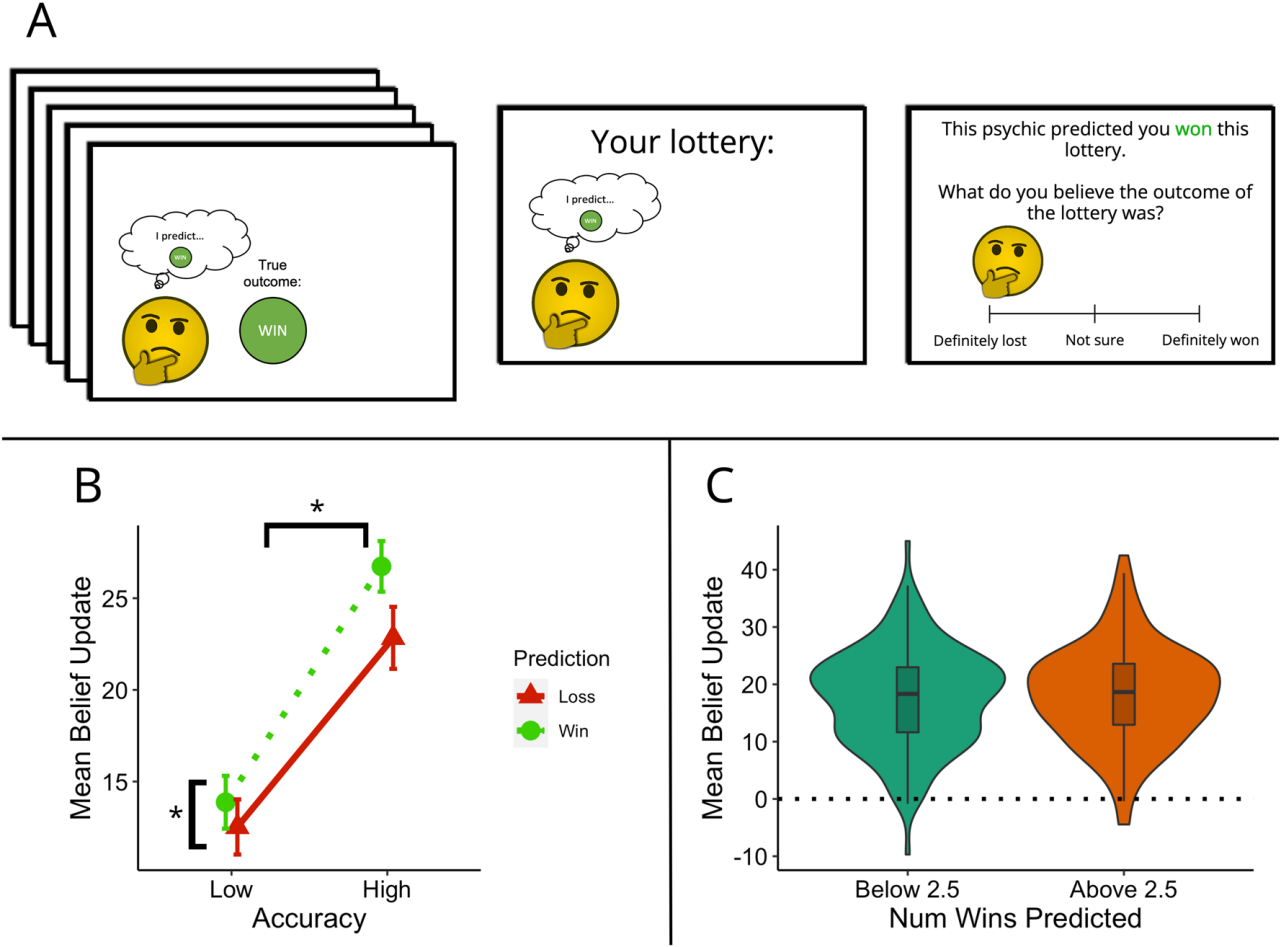
(**A**) Paradigm for Experiment 2. On each trial, participants were first shown a series of five example predictions made by the psychic. The true outcome of each of these lotteries was also shown. The psychic then provided a prediction of the participant’s own lottery, for which the true outcome was not shown. Participants were then asked to rate their beliefs about the outcome of their lottery on a continuous scale. (**B**) Mean magnitude of belief update following positive and negative predictions made by psychics with high and low accuracy. Error bars denote the standard error of the mean. Greater belief updates were made following predictions from more accurate psychics. Participants also updated their beliefs more in response to positive predictions than negative ones. Asterisks denote significant main effects of accuracy (high/low) and prediction (win/loss). (**C**) Distribution of mean belief updates following predictions from psychics with below or above the average number of positive example predictions.

To determine whether our results were attributable to the pursuit of hedonic goals or to differences in perceived credibility due to attentional or weighting factors, we conducted a follow-up study in a separate group of participants. In each trial, participants were shown a set of five example predictions made by a single psychic (see Fig. 3A). These predictions could again vary in how accurate and how positive they were (see “Methods”). The psychic then predicted the outcome of the participant’s lottery. Importantly, in this experiment, psychics who provided biased example predictions were not more likely to provide biased predictions for the participant’s lottery. This removed the confound of differences in the performance of the different psychics when predicting the participants’ own lotteries. Following this, participants provided a rating of what they believed the outcome of their lottery was on a continuous scale from 0 (definitely lost) to 100 (definitely won). Belief updates were calculated as the difference between the participant’s belief rating and the point of indifference (50 on the response scale, which is the most plausible belief to hold in this lottery before observing any prediction) relative to the direction of the psychic's prediction. For example, if the psychic predicted the participant would lose their lottery and the participant rated their beliefs as 10, this would be quantified as a 40-point update, while a rating of 60 would be quantified as a − 10-point update. Alternatively, if the psychic predicted a winning outcome, a response of 90 would constitute a 40-point update, while a response of 40 would be quantified as a -10-point update.

#### Accuracy of sources, positive predictions and credibility

First, we investigated the relationship between the accuracy of the sources and what they predicted for the participant’s own lotteries. We ran a 2 $\times$ 2 two-way repeated measures ANOVA with within-subjects factors of Accuracy (high or low) and Prediction for participant’s lottery (win or loss). This showed significant main effects of Accuracy, *F*(153) = 248.48, *p* < 0.001, $\eta _{p}^{2}$ = 0.62, and Prediction for participant’s lottery, *F*(153) = 20.89, *p* < 0.001, $\eta _{p}^{2}$ = 0.12, indicating that participants updated their beliefs more following predictions made by high-accuracy sources than low-accuracy sources. The interaction effect was also significant, *F*(153) = 9.98, *p* = 0.002, $\eta _{p}^{2}$ = 0.06, with larger differences in belief updates between positive and negative predictions found for high accuracy psychics (Fig. 3B).

#### Positively-biased sources and credibility

Next, we investigated whether sources that made more positive predictions across the five example predictions were perceived as more credible. Note that in this experiment, this positivity bias in the example predictions was dissociated from the valence of the final prediction of the participants’ lottery, which allowed us to separate out these factors. Analysis suggested that belief update magnitude did not differ depending on the number of “win” predictions made by the psychics during the example lotteries (Fig. 3C). A paired-samples t-test indicated that belief updates were not larger for the more positive half of psychics relative to the more negative half, *t*(153) = 1.32, *p* = 0.18.

To understand this further, we constructed two computational models. In the *Full Model*, both accuracy and positivity of psychics’ example predictions were used to predict the magnitude of participants’ belief updates. In the *No Positivity Model*, only accuracy was used to predict belief updates. Both models also included individual-level free parameters governing the asymmetry of beliefs following positive and negative predictions ($k_{Sym}$) and constant mean level of belief updating ($k_{Cons}$; see “Methods” for details). The $k_{Sym}$ parameter captures variance from both asymmetry in the process of updating and from asymmetry in belief starting-points (consistent with an optimism bias). While these processes are distinct, fitting parameters to each would lead to an unrecoverable model, as changes to each predict the same patterns of belief updates. Using the same model fitting procedures as Experiment 1, the models were fit hierarchically, such that the distributions of individual-level parameters were governed by a set of group-level hyperparameters.

Model comparisons suggested that the *No Positivity Model* provided the best predictions of the data (Table 2). This model comprised the entirety of the optimal weighted combination of model predictions, indicating that the inclusion of positivity in the *Full Model* did not improve model predictions.

**Table 2:** Experiment 2 model fit statistics. Estimated expected log-posterior density (elpd), Watanabe–Akaike Information Criterion (WAIC), Bayesian stacking weights, and the proportion of participants whose data was best predicted by each model. Larger positive values of elpd and smaller values of WAIC indicate better predictive performance. The difference from the best-fitting model is represented as $\Delta {\hat{elpd}}$.

Model	${\hat{elpd}}$ (S.E.M)	$\Delta {\hat{elpd}}$ (S.E.M)	WAIC (S.E.M)	Bayesian weight	Prop. best predictor
Full model	− 216 (324)	− 2.44 (1.97)	510.82 (206.32)	< 0.001	0.497
No positivity model	− 214 (324)	–	508.93 (206.60)	> 0.999	0.503

Bayesian stacking weights correspond to the weights assigned to the predictions of each model that maximise the log predictive density of future data

However, the *Full Model* provided the best fitting predictions of nearly half of the participants’ data (Table 2). This may either be because, for a subset of participants, positivity affected the perceived credibility of psychics, or because estimated values of the additional parameter ($k_{Pos}$, corresponding to the extent to which positivity affected belief updates) were small enough such that the more complex Full Model mimicked the performance of the simpler No Positivity Model. To further investigate which of these explanations is most plausible, we examined the posterior distributions of the accuracy and positivity hyperparameters $\mu _{k_{Acc}}$ and $\mu _{k_{Pos}}$. To assess whether the posterior estimates were meaningfully different from 0, we constructed a region of practical equivalence (ROPE)^48^ for each. The bounds of the ROPE were set at the point at which the maximum change in the independent variable (i.e., the difference between five positive example predictions vs. five negative example predictions and the difference between four accurate vs. three accurate example predictions) produced a 0.1 standard deviation change in the dependent variable. As per recommendations, a parameter whose entire 95% HDI is contained within the ROPE is treated as not meaningfully different from zero^48^.

**Figure 4:**
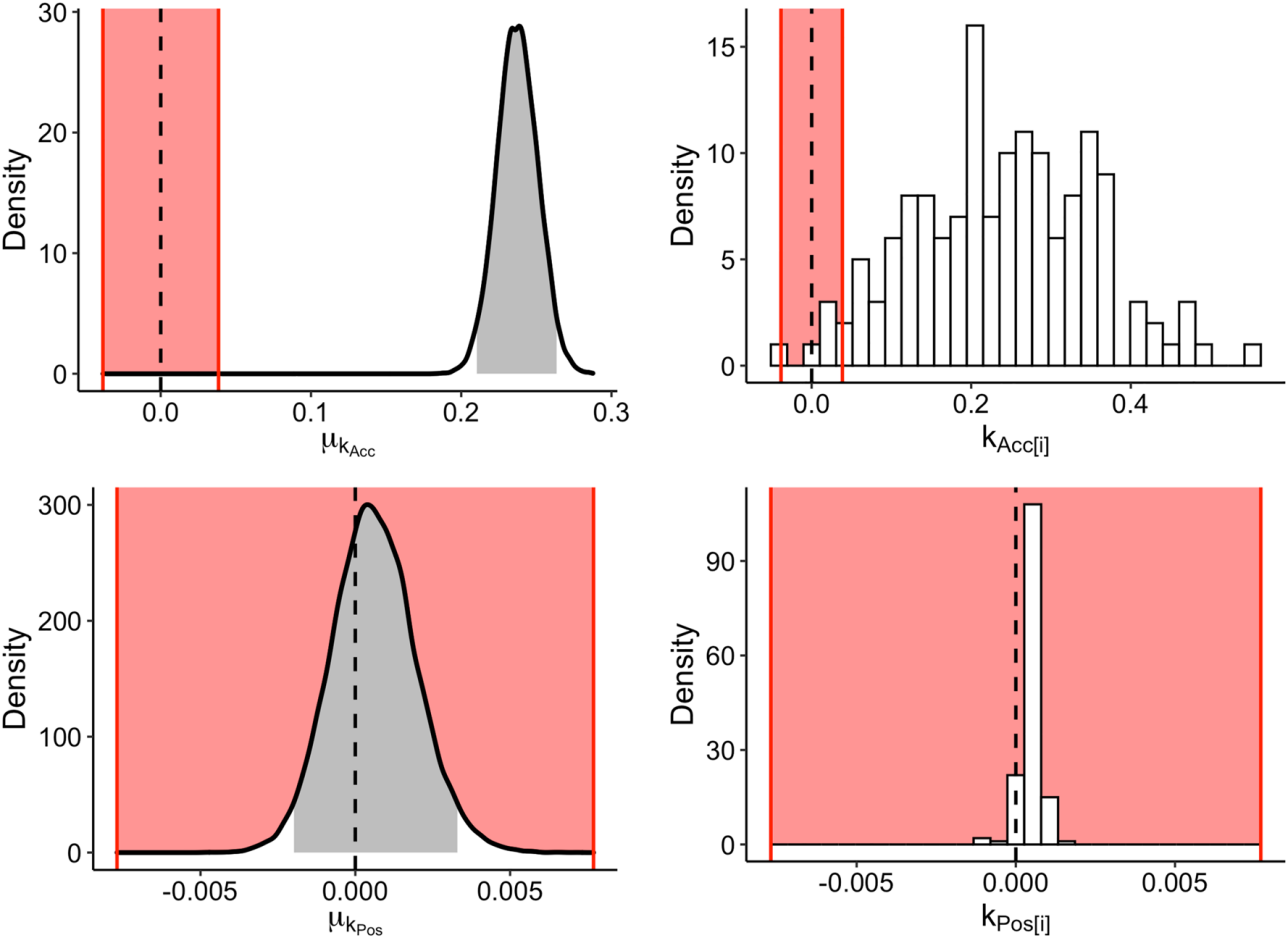
Left column: Posterior distribution of the hyperparameters $\mu _{k_{Acc}}$ and $\mu _{k_{Pos}}$ obtained from fitting the *Full Model*. The grey shaded areas denote the 95% highest density intervals. Right column: Histograms of individual parameter point estimates of $k_{Acc}$ and $k_{Pos}$ for each participant. In all plots, the red shaded areas indicate the region of practical equivalence, within which parameter values can be inferred to be meaningfully equivalent to zero.

Results showed that the $\mu _{k_{Acc}}$ accuracy hyperparameter was meaningfully different from zero, whereas the $\mu _{k_{Pos}}$ positivity hyperparameter was not (Fig. 4). This indicates that positively-biased psychics were not seen as more credible, with the *Full Model* providing the best fit to some participants because it mimicked the performance of the simpler *No Positivity Model*. These results therefore suggest that positive prediction only mattered for belief updates when these were related to the final prediction for the participant’s own lottery. There was no evidence, however, that overall more positive sources (across all example predictions) were also perceived as more credible (i.e., led to stronger belief updates).

It is therefore reasonable to conclude that the preference for positively-biased information sources found in Experiment 1 was likely due to hedonic goals rather than to a change in credibility perception. The observed increases in confidence associated with positively-biased information-seeking in Experiment 1, as well as the larger belief updates in Experiment 2, are in turn more likely to be attributable to asymmetrical updating of beliefs such that positive predictions are believed more readily than negative ones.

## Discussion

In this study, we aimed to examine the role of the hedonic value of information on information search and belief formation. Experiment 1 revealed that, while accuracy was the strongest motivator of information preference, a large proportion of our participants also showed a preference for positively-biased information despite being presented with a clear, unambiguous measure of source accuracy. Of these participants, the majority showed a willingness to seek information from positively-biased sources even at the expense of a more accurate alternative. Those seeking positively-biased information tended to form more positive beliefs about the outcomes they were informed about. Importantly, biased information seekers held higher confidence in their beliefs. In Experiment 2, we showed that the pursuit of biased information was not attributable to the perception of biased sources as being more credible, despite positive predictions about one’s own outcomes producing enhanced belief updates beyond the sources’ accuracy^31–33^. Together, these findings reveal the hedonic value of information to be a key contributor to information source selection and consequent belief formation.

Participants not only sampled positively-biased information more readily, but also held the beliefs they formed on the basis of the predictions yielded by positively-biased sources in higher confidence. Previous work has shown that individuals with extreme beliefs tend to have higher confidence in their beliefs^27,28^. Here, we showed that those with the highest confidence in their beliefs about the world-state were those who were biased towards information sources that were positively-biased, rather than those sampling from more objectively accurate sources. In addition, data from Experiment 2 revealed that this association between hedonic bias and confidence was not because biased predictors are necessarily perceived as more credible. Instead, this effect of biased information on confidence may be explained by the tendency for positive information to be more readily accepted due to asymmetry in belief updating^31–33^ and to optimism bias^46,47^. As the current experimental design cannot distinguish between these processes, further research is required to identify which contributes to this behaviour. Together, our results build on previous work by showing that hedonic bias in information-seeking behaviour is a key contributor to extreme, high-confidence beliefs.

Another striking aspect of our results is the differential perception of self-relevant versus other-relevant lottery predictions. In Experiment 2, predicting positive outcomes on other lotteries did not increase the perceived credibility of a psychic. However, positive predictions about the outcome of one’s own lottery were believed more strongly than negative predictions. These results are in line with research showing that positively-valenced information is more readily believed when it is self-relevant.^49^. This processing difference has been shown to contribute to the formation of positively-biased self-relevant beliefs^50–52^. We extend on these findings to highlight the active role of biased information-search as a contributor to this phenomenon.

Our results not only support previous findings that individuals prefer positively-valenced information^17–19^, but also demonstrate that this preference persists even when more objectively accurate sources of information are available. Furthermore, the hedonic value of the information in our study was low (given the low stakes involved) relative to the many real-world scenarios in which information is typically sought^12,19^. Information-seeking behaviour increases in situations in which the perceived relevance of the subject-matter is greater^4,53^. The present study therefore provides compelling evidence for the influence of hedonic value on information preference, even when that information is of little emotional or ideological relevance.

The present findings highlight important avenues for future research. These results demonstrate that both accuracy and expected valence are important determinants of the subjective value of an information source. However, due to the experimental design used, in which each combination of accuracy and positivity was not compared with all other combinations, the interaction between the two cannot be examined. Future studies may aim to investigate whether these two factors interact to determine the subjective value of information. Further, while this study demonstrates one mechanism through which the pursuit of hedonic goals may shape information-seeking behaviour and beliefs, the impact of this contribution relative to other factors remains unknown. Another factor to consider is Bayesian rationalism; the extent to which prior beliefs influence our perception of the credibility of information sources. Characterising the relative contributions of each to information-seeking behaviour will allow for the design of more optimal interventions for combatting confirmation bias, targeted either at reducing the role of hedonism (e.g., by focusing on reducing the association between congenial information and hedonic value) or of Bayesian rationalism (e.g., by focusing on broadening prior beliefs).

The present study provides clear evidence for the role of the hedonic pursuit of desirable affective states in information selection. We further expanded this by elucidating the cognitive mechanisms underlying this behaviour and its subsequent consequences on beliefs. Seeking positively-biased information led to the formation of more positive beliefs, which were held with greater confidence than those based on less biased, more accurate information. In order to combat confirmation bias and reduce the polarisation of beliefs, we must gain a better understanding of the other factors contributing to this behaviour. By doing so, we will be better able to design interventions to reduce the extent to which we pursue information that is consistent with our beliefs.

## Methods

### Experiment 1

#### Participants

Using the online recruitment platform Prolific^54^, we recruited a sample of 225 participants (94 female, 130 male, 1 other, *M* = 31.39, *SD* = 11.30). Of these, we excluded 39 for failing a post-experiment check (see below) and a further 28 were excluded for self-reported random responses. The remaining sample of 158 participants (67 female, 91 male) were aged between 18 and 69 (*M* = 31.70, *SD* = 11.32). For their participation, participants received a reimbursement of either AUD $8 or their total winnings from the experiment, whichever was largest. In both experiments, informed consent was provided by all participants, and research was conducted in accordance with the Declaration of Helsinki. All study protocols were approved by The University of Melbourne Human Research Ethics Committee (ID 23253).

#### Procedure

All stimuli were presented using the jsPsych 6.3.1 library^55^. Before commencing the experiment, participants were presented with comprehensive written instructions for the task. Participants were instructed that, on each trial, they would be participating in a lottery in which they could either win 20c or win nothing. The outcome of each lottery would not be revealed to them, but they would be allowed to see the prediction of one of two computer-generated “psychics” (represented by standard emojis; Unicode U+1F914) of the outcome of their lottery. To help them choose which psychic’s prediction to view, they would first be shown the psychics making a series of five predictions of other, irrelevant lotteries. After each trial, the psychics would be swapped for new ones before the commencement of the next lottery.

Following these instructions, participants completed a series of questions to check their understanding of the task. Importantly, the questions reiterated that (1) their chosen psychic’s prediction did not necessarily determine whether they won or lost their lottery - they merely made a prediction; (2) The psychics would be swapped for new ones after each of the participant’s lotteries. Hence, they should only base their choice of psychic on the five example predictions immediately preceding their lottery; and (3) The outcomes of the five example lotteries did not affect the participant’s winnings; they were simply to help the participant to decide which psychic’s prediction they would like to see. Feedback was provided for each response given to the questions. Upon completion of the task, the understanding check questions were repeated. Any participant that failed to respond correctly to all three questions after completing the experiment was excluded from analyses.

On each trial, participants were first shown both psychics side-by-side making a series of predictions about the outcomes of a set of five lotteries. Each prediction was preceded by a 1.25 s delay, before psychics’ predictions were shown for 1.25 s, before the true outcome of the lottery was shown for a further 1.5 s. After this sequence was repeated five times, the participant was prompted to select either the left of right psychic using the ‘F’ or ‘J’ key, corresponding to the left and the right psychic, respectively. After their selection, there was an inter-stimulus interval of 1.25 s, following which the participant’s chosen psychic’s prediction of their lottery was shown for 1.25 s. Crucially, the probability of a psychic predicting a “win” outcome when chosen by the participant was proportional to their bias towards predicting winning outcomes in the five example trials. This, however, was not explicitly mentioned to participants. The true outcome was not shown for participants’ lotteries.

After each block of 16 trials, participants were prompted to estimate how many of the past 16 lotteries they had won and provide a confidence rating of their estimate, ranging from ‘Not confident at all’ (0) to ‘Extremely confident’ (100). Slider starting position was randomised for both questions, and the participant was required to move them prior to submitting their response.

At the end of the experiment, participants rated their agreement with three statements on a five-point scale (‘Strongly Disagree’ to ‘Strongly Agree’): (1) “I chose which psychic’s prediction to view at random”, (2) “I chose to view the predictions of the psychic who made the most accurate predictions”, (3) “I chose to view the predictions of the psychic who predicted “win” outcomes most often”. Participants who agreed most strongly with the statement indicating random responses were excluded from analyses. Following this, participants were presented with an open-ended prompt: “How did you choose which psychic’s prediction to view?”. Before answering any of these questions, participants were assured that their responses would not impact their payment for completing the study.

#### Trial structure

Trials varied on three key characteristics regarding the five example predictions: the number of “true wins” in the sequence (i.e., on how many of the example predictions was the true outcome of the lottery a “win”), the number of accurate predictions made by each psychic (i.e., on how many of the five example predictions did each psychic accurately predict the true outcome), and the number of positive predictions made by each psychic (i.e., on how many of the five example predictions did the psychic predict a “win”). To ensure that inverse inference was never the optimal strategy, the minimum accuracy of both psychics was limited at three out of five.

Given these restrictions, the number of possible combinations of trials was 56. To limit experiment length and ensure each combination could be repeated within participants, a sample of 16 combinations, representative of the full distribution, were manually selected (see Supplementary Materials, Table A6). Each trial was presented once per block. Within each block, trial order and the position of the two psychics was randomised. There were four blocks of trials in total, each of which took approximately 6.5 min to complete.

#### Computational modelling

We constructed a series of computational models to characterise the factors driving information preference. The models were fit using Hamiltonian Monte Carlo sampling as implemented in Stan^56^. Each model was fit using four parallel chains with a warm-up period of 1500 samples each followed by 5000 samples drawn from the converged chains. To reduce correlation between parameters, all variables were centred prior to modelling^57^.

The first model (*Accuracy & Positivity Model*) operationalised the hypothesis that participants value an information source based both on how accurate it is and how likely it is to yield positively-valenced information. Within this model the value of an information source *X* is given by a linear, weighted combination of the number of accurate predictions made by the source and the number of “wins” the source predicted. The weights assigned to each are subject-level free parameters $k_{Acc}$ and $k_{Pos}$, respectively (Eq. 1).

1 $$\begin{aligned} \mu _{V[X,i]} = k_{Acc[i]} \, nAcc_{[X]} + k_{Pos[i]} \, nPos_{[X]}. \end{aligned}$$

The value of each competing source were then entered into a softmax function (Eq. 2) to determine the predicted probability of choosing each option.

2 $$\begin{aligned} Pr(X) = \frac{e^{\beta \cdot V(X)}}{e^{\beta \cdot V(X)} + e^{\beta \cdot V(Y)}}, \beta \in [0, 5]. \end{aligned}$$

Within the softmax function, the competing sources are represented as *X* and *Y*, while $\beta$ represents the subject-specific inverse temperature parameter that determines the stochasticity of participants’ responses. To estimate the free parameters in this model, we adopted a hierarchical Bayesian estimation strategy which assumes that each participant’s parameters are drawn from joint group-level distributions such that the free parameters for subject *i* are drawn from the prior distributions shown in Eq. (3).

3 $$\begin{aligned} \begin{aligned} k_{Acc}&\sim \text {Normal}(\mu _{k_{Acc}}, \sigma _{k_{Acc}}) \\ k_{Pos}&\sim \text {Normal}(\mu _{k_{Pos}}, \sigma _{k_{Pos}}) \\ \beta&\sim \text {Normal}(\mu _{\beta }, \sigma _{\beta }), \; \in [0, 5]. \end{aligned} \end{aligned}$$

The prior distributions for these parameters were weakly informative, preventing the model from adopting unreasonable parameter estimates (Eq. 4).

4 $$\begin{aligned} \begin{aligned} \mu _{k_{Acc}}, \mu _{k_{Pos}}&\sim \text {Normal}(0, 1) \\ \mu _{\beta }&\sim \text {Normal}(0, 2) \\ \sigma _{k_{Acc}}, \sigma _{k_{Pos}}, \sigma _{\beta }&\sim \text {Gamma}(1, 0.5). \end{aligned} \end{aligned}$$

The *Accuracy Only Model* operationalised the hypothesis that only accuracy was important for participants when determining which information source to choose. This model used the same functional structure as the *Accuracy & Positivity Model*, but with positivity removed as a predictor of the value of the information source. The full model specification and priors are listed in Eq. (5).

5 $$\begin{aligned} \begin{aligned} \mu _{V[X,i]}&= k_{Acc[i]} \, nAcc_{[X]} \\ k_{Acc}&\sim \text {Normal}(\mu _{k_{Acc}}, \sigma _{k_{Acc}}) \\ \beta&\sim \text {Normal}(\mu _{\beta }, \sigma _{\beta }), \; \in [0, 5] \\ \mu _{k_{Acc}}&\sim \text {Normal}(0, 1) \\ \mu _{\beta }&\sim \text {Normal}(0, 2) \\ \sigma _{k_{Acc}}, \sigma _{\beta }&\sim \text {Gamma}(1, 0.5). \end{aligned} \end{aligned}$$

Similarly, the *Positivity Only Model* operationalised the hypothesis that participants only utilised the number of wins each source predicted when choosing between them. Similarly, this model used the same structure as the Accuracy & Positivity Model, but with accuracy removed as a predictor of the value of the information source. The full model specification and priors are listed in Eq. (6).

6 $$\begin{aligned} \begin{aligned} \mu _{V[X,i]}&= k_{Pos[i]} \, nPos_{[X]} \\ k_{Pos}&\sim \text {Normal}(\mu _{k_{Pos}}, \sigma _{k_{Pos}}) \\ \beta&\sim \text {Normal}(\mu _{\beta }, \sigma _{\beta }), \; \in [0, 5] \\ \mu _{k_{Pos}}&\sim \text {Normal}(0, 1) \\ \mu _{\beta }&\sim \text {Normal}(0, 2) \\ \sigma _{k_{Pos}}, \sigma _{\beta }&\sim \text {Gamma}(1, 0.5). \end{aligned} \end{aligned}$$

Finally, the *Accuracy & Positivity Tiebreak Model* operationalised the hypothesis that accuracy of information was the only determining factor for participants when choosing between sources, with the number of positive predictions used only as a tiebreaker when the two sources were equally accurate. This was achieved by using the same structure as the Accuracy & Positivity Model, but multiplying the $k_{Pos}$ parameter with the binary variable *EQ* which was equal to 0 when the psychics had the same number of correct predictions and 1 on trials in which the psychics were equally accurate (Eq. 7).

7 $$\begin{aligned} \begin{aligned} \mu _{V[X,i]}&= k_{Acc[i]} \, nAcc_{[X]} + k_{Pos[i]} \, nPos_{[X]} \, EQ_{[X]}\\ k_{Acc}&\sim \text {Normal}(\mu _{k_{Acc}}, \sigma _{k_{Acc}}) \\ k_{Pos}&\sim \text {Normal}(\mu _{k_{Pos}}, \sigma _{k_{Pos}}) \\ \beta&\sim \text {Normal}(\mu _{\beta }, \sigma _{\beta }), \; \in [0, 5] \\ \mu _{k_{Acc}}, \mu _{k_{Pos}}&\sim \text {Normal}(0, 1) \\ \mu _{\beta }&\sim \text {Normal}(0, 2) \\ \sigma _{k_{Acc}}, \sigma _{k_{Pos}}, \sigma _{\beta }&\sim \text {Gamma}(1, 0.5). \end{aligned} \end{aligned}$$

### Experiment 2

#### Participants

We recruited an independent sample of 180 participants using Prolific^54^ (63 female, 115 male, 2 other, *M* = 34.21, *SD* = 12.38). Of the recruited participants, 31 were excluded for failing a post-experiment understanding check. The remaining sample of 149 participants (53 female, 94 male, 2 other) were aged between 18 and 69 (*M* = 33.51, *SD* = 11.98). For their participation, participants received a reimbursement of either AUD $5.30 or their total winnings from the experiment, whichever was largest.

#### Procedure

The procedure was similar to Experiment 1. However, in this Experiment, only one psychic made example predictions on each trial. That psychic would then proceed to predict the outcome of the participant’s own lottery. Following this, the participant provided a confidence rating of what they believed the outcome of their lottery to be. This rating was on a continuous scale from 0 (definitely lost) to 100 (definitely won). Participants were instructed that different psychics would be making the predictions on each trial, and each psychic’s predictions were entirely independent. The same post-instructions questions as in Experiment 1 were used as an understanding check and a means to exclude participants who did not comply with task requirements.

#### Trial structure

To limit experiment length and ensure each combination could be repeated within participants, only trials in which the true wins were either 2 or 3 out of 5, and psychic accuracy was 3 or 4 out of 5 were used (see Supplementary Materials, Table A2). Each trial was repeated four times. On two of the repetitions, the psychic predicted a win for the participant’s lottery; on two they predicted a loss. Trials were split across four blocks, with the order of trials pseudo-randomised such that two repetitions of each trial occurred within the first two blocks and two occurred in the last two blocks. Each block took approximately 5 min to complete.

#### Computational modelling

We constructed two computational models to characterise participants’ belief updating. All models were fit using the same protocol as described in Experiment 1.

Within the *Full Model* model, participants’ beliefs were modelled using five subject-level free parameters (see Eq. 8). The first, $k_{Cons}$, corresponds to the constant, baseline level of belief update for participant. The $k_{Acc}$ and $k_{Pos}$ parameters dictate the respective extent to which the accuracy and positivity of the psychic’s example predictions affect belief update magnitude. The $k_{Sym}$ parameter corresponds to the asymmetry in update magnitude following a positive (rather than negative) prediction on the participant’s own lottery. This is achieved by multiplying $k_{Sym}$ by the binary *Dir*, which takes the value of 1 on trials in which the psychic predicted the participant would win, or 0 on trials in which they predicted a loss. Variance in $k_{Sym}$ reflects the combination of optimism bias (differences in belief starting-point) and the asymmetry of belief updates for positive and negative outcomes. The final free parameter was the $\sigma _{U[i]}$ parameter, corresponding to the standard deviation of the participant’s bids around the predicted mean.

8 $$\begin{aligned} \begin{aligned} U[X,i]&\sim \text {Normal}(\mu _{U[X,i]}, \sigma _{U[i]}) \\ \mu _{U[X,i]}&= (k_{Cons} + k_{Acc[i]} \, nAcc_{[X]} + k_{Pos[i]} \, nPos_{[X]}) \cdot (1 + (k_{Sym} - 1) \cdot Dir) \\ \sigma _{U[i]}&\sim \text {Gamma}(1, 0.5). \end{aligned} \end{aligned}$$

A hierarchical Bayesian estimation strategy was again employed such that the free parameters for subject *i* are drawn from the prior distributions (Eq. 9).

9 $$\begin{aligned} \begin{aligned} k_{Cons}&\sim \text {Normal}(\mu _{k_{Cons}}, \sigma _{k_{Cons}}) \\ k_{Acc}&\sim \text {Normal}(\mu _{k_{Acc}}, \sigma _{k_{Acc}}) \\ k_{Pos}&\sim \text {Normal}(\mu _{k_{Pos}}, \sigma _{k_{Pos}}) \\ k_{Sym}&\sim \text {Normal}(\mu _{k_{Sym}}, \sigma _{k_{Sym}}). \end{aligned} \end{aligned}$$

Weakly informative prior distributions were employed to prevent the model from adopting unreasonable parameter estimates (Eq. 10).

10 $$\begin{aligned} \begin{aligned} \mu _{k_{Cons}}, \mu _{k_{Acc}}, \mu _{k_{Pos}}, log(\mu _{k_{Sym}})&\sim \text {Normal}(0, 1) \\ \sigma _{k_{Cons}}, \sigma _{k_{Acc}}, \sigma _{k_{Pos}}, \sigma _{k_{Sym}}&\sim \text {Gamma}(1, 0.5). \end{aligned} \end{aligned}$$

A comparison model was also constructed. In the *No Positivity Model*, the same parameters were used with the exception of the $k_{Pos}$ parameter, which was removed. The model specification and priors are presented in equation 11, for completeness.

11 $$\begin{aligned} \begin{aligned} U[X,i]&\sim \text {Normal}(\mu _{U[X,i]}, \sigma _{U[i]}) \\ \mu _{U[X,i]}&= (k_{Cons} + k_{Acc[i]} \, nAcc_{[X]}) \cdot (1 + (k_{Sym} - 1) \cdot Dir) \\ \sigma _{U[i]}&\sim \text {Gamma}(1, 0.5) \\ k_{Cons}&\sim \text {Normal}(\mu _{k_{Cons}}, \sigma _{k_{Cons}}) \\ k_{Acc}&\sim \text {Normal}(\mu _{k_{Acc}}, \sigma _{k_{Acc}}) \\ k_{Sym}&\sim \text {Normal}(\mu _{k_{Sym}}, \sigma _{k_{Sym}}) \\ \mu _{k_{Cons}}, \mu _{k_{Acc}}, log(\mu _{k_{Sym}})&\sim \text {Normal}(0, 1) \\ \sigma _{k_{Cons}}, \sigma _{k_{Acc}}, \sigma _{k_{Sym}}&\sim \text {Gamma}(1, 0.5). \end{aligned} \end{aligned}$$

## Supplementary Information

Supplementary Information.

## Data Availability

Requests for the data can be sent via email to the corresponding author.

## References

[1] Coibion, O., Gorodnichenko, Y. & Weber, M. Political Polarization and Expected Economic Outcomes. Tech. Rep., National Bureau of Economic Research, Cambridge (2020). 10.3386/W28044.

[2] Jenkins-Smith HC (2020). Partisan asymmetry in temporal stability of climate change beliefs. Nat. Clim. Change.

[3] Jamieson K, Cappella J (2010). Echo Chamber: Rush Limbaugh and the Conservative Media Establishment.

[4] Cappella JN, Kim HS, Albarracín D (2014). Selection and transmission processes for information in the emerging media environment: Psychological motives and message characteristics. Med. Psychol..

[5] Hart W (2009). Feeling validated versus being correct: A meta-analysis of selective exposure to information. Psychol. Bull..

[6] Peterson E, Iyengar S (2021). Partisan gaps in political information and information-seeking behavior: Motivated reasoning or cheerleading?. Am. J. Polit. Sci..

[7] Slater MD (2007). Reinforcing spirals: The mutual influence of media selectivity and media effects and their impact on individual behavior and social identity. Commun. Theory.

[8] Stroud, N. J. Selective exposure theories. In *The Oxford Handbook of Political Communication*, vol. 1. (eds. Kenski, K. & Jamieson, K. H.) (Oxford University Press, 2017) 10.1093/OXFORDHB/9780199793471.013.009_UPDATE_001.

[9] Iyengar S, Hahn KS (2009). Red media, blue media: Evidence of ideological selectivity in media use. J. Commun..

[10] O’Connor C, Weatherall JO (2018). Scientific polarization. Eur. J. Philos. Sci..

[11] Stroud NJ (2010). Polarization and partisan selective exposure. J. Commun..

[12] Pennycook G, Rand DG (2021). The psychology of fake news. Trends Cogn. Sci..

[13] Cook J, Lewandowsky S (2016). Rational irrationality: Modeling climate change belief polarization using Bayesian networks. Top. Cogn. Sci..

[14] Druckman JN, McGrath MC (2019). The evidence for motivated reasoning in climate change preference formation. Nat. Clim. Chang..

[15] Perfors, A. & Navarro, D. J. Why do echo chambers form? The role of trust, population heterogeneity, and objective truth. In *Proceedings of the 41st Annual Conference of the Cognitive Science Society*, 918–923 (Cognitive Science Society, 2019).

[16] Jonas, E., Graupmann, V. & Frey, D. The influence of mood on the search for supporting versus conflicting information: Dissonance reduction as a means of mood regulation? 10.1177/0146167205276118 (2006). 10.1177/0146167205276118 16317184

[17] Charpentier CJ, Bromberg-Martin ES, Sharot T (2018). Valuation of knowledge and ignorance in mesolimbic reward circuitry. Proc. Natl. Acad. Sci. U.S.A..

[18] Jiwa M, Cooper PS, Chong TT, Bode S (2021). Choosing increases the value of non-instrumental information. Sci. Rep..

[19] Sharot T, Sunstein CR (2020). How people decide what they want to know. Nat. Hum. Behav..

[20] Bromberg-Martin ES, Sharot T (2020). The value of beliefs. Neuron.

[21] Bennett D, Sutcliffe K, Tan NPJ, Smillie LD, Bode S (2021). Anxious and obsessive-compulsive traits are independently associated with valuation of noninstrumental information. J. Exp. Psychol. Gen..

[22] Goh AX, Bennett D, Bode S, Chong TT (2021). Neurocomputational mechanisms underlying the subjective value of information. Commun. Biol..

[23] Liuzzi L (2022). Magnetoencephalographic correlates of mood and reward dynamics in human adolescents. Cereb. Cortex.

[24] Keren H (2021). The temporal representation of experience in subjective mood. eLife..

[25] Suhay E, Erisen C (2018). The role of anger in the biased assimilation of political information. Polit. Psychol..

[26] Epley N, Gilovich T (2016). The mechanics of motivated reasoning. J. Econ. Perspect..

[27] Toner K, Leary MR, Asher MW, Jongman-Sereno KP (2013). Feeling superior is a bipartisan issue: Extremity (not direction) of political views predicts perceived belief superiority. Psychol. Sci..

[28] Harris EA, Van Bavel JJ (2021). Preregistered replication of "feeling superior is a bipartisan issue: Extremity (not direction) of political views predicts perceived belief superiority". Psychol. Sci..

[29] Lavine H, Lodge M, Freitas K (2005). Threat, authoritarianism, and selective exposure to information. Polit. Psychol..

[30] Zmigrod L (2020). The role of cognitive rigidity in political ideologies: Theory, evidence, and future directions. Curr. Opin. Behav. Sci..

[31] Eil D, Rao JM (2011). The good news-bad news effect: Asymmetric processing of objective information about yourself. Am. Econ. J. Microecon..

[32] Korn CW, Prehn K, Park SQ, Walter H, Heekeren HR (2012). Positively biased processing of self-relevant social feedback. J. Neurosci..

[33] Sharot T, Korn CW, Dolan RJ (2011). How unrealistic optimism is maintained in the face of reality. Nat. Neurosci..

[34] Metzger MJ, Hartsell EH, Flanagin AJ (2015). Cognitive dissonance or credibility? A comparison of two theoretical explanations for selective exposure to partisan news. Commun. Res..

[35] Magnusson, M., Andersen, M. R., Jonasson, J. & Vehtari, A. Leave-one-out cross-validation for Bayesian model comparison in large data. In *International Conference on Artificial Intelligence and Statistics*, 341–351 (2020).

[36] Browne MW (2000). Cross-validation methods. J. Math. Psychol..

[37] Roberts DR (2017). Cross-validation strategies for data with temporal, spatial, hierarchical, or phylogenetic structure. Ecography.

[38] Vehtari A, Lampinen J (2002). Bayesian model assessment and comparison using cross-validation predictive densities. Neural Comput..

[39] Yao Y, Vehtari A, Simpson D, Gelman A (2018). Using stacking to average Bayesian predictive distributions (with discussion). Bayesian Anal..

[40] Watanabe, S. Asymptotic equivalence of Bayes cross validation and widely applicable information criterion in singular learning theory. *J. Mach. Learn. Res.* 1004.2316 (2010).

[41] Gelman A, Hwang J, Vehtari A (2014). Understanding predictive information criteria for Bayesian models. Stat. Comput..

[42] Kelly CA, Sharot T (2021). Individual differences in information-seeking. Nat. Commun..

[43] Kobayashi K, Ravaioli S, Baranès A, Woodford M, Gottlieb J (2019). Diverse motives for human curiosity. Nat. Hum. Behav..

[44] Bates, D., Mächler, M., Bolker, B. & Walker, S. Fitting linear mixed-effects models using lme4. *J. Stat. Softw*. 10.18637/jss.v067.i01 (2015).

[45] Kaanders P, Sepulveda P, Folke T, Ortoleva P, De Martino B (2022). Humans actively sample evidence to support prior beliefs. eLife..

[46] Sharot T, Riccardi AM, Raio CM, Phelps EA (2007). Neural mechanisms mediating optimism bias. Nature.

[47] Sharot T (2011). The optimism bias. Curr. Biol..

[48] Kruschke JK, Liddell TM (2018). The Bayesian new statistics: Hypothesis testing, estimation, meta-analysis, and power analysis from a Bayesian perspective. Psychon. Bull. Rev..

[49] Sharot T, Garrett N (2016). Forming beliefs: Why valence matters. Trends Cogn. Sci..

[50] Kuzmanovic B, Rigoux L (2017). Valence-dependent belief updating: Computational validation. Front. Psychol..

[51] Chowdhury R, Sharot T, Wolfe T, Düzel E, Dolan RJ (2014). Optimistic update bias increases in older age. Psychol. Med..

[52] Moutsiana C (2013). Human development of the ability to learn from bad news. Proc. Natl. Acad. Sci. U.S.A..

[53] Charpentier CJ (2022). Anxiety increases information-seeking in response to large changes. Sci. Rep..

[54] Palan S, Schitter C (2018). Prolific.ac A subject pool for online experiments. J. Behav. Exp. Finance.

[55] de Leeuw JR (2014). jsPsych: A JavaScript library for creating behavioral experiments in a Web browser. Behav. Res. Methods.

[56] Carpenter, B. *et al.* Stan: A probabilistic programming language. *J. Stat. Softw.*10.18637/jss.v076.i01 (2017). 10.18637/jss.v076.i01 PMC9788645 36568334

[57] McElreath, R. *Statistical rethinking: A Bayesian Course with Examples in R and Stan* (Chapman and Hall/CRC, 2018).

