# Supplementary material for: Hedonism as a motive for information search: biased information-seeking leads to biased beliefs
Source: Sci Rep. 2023 Feb 6;13:2086. doi: 10.1038/s41598-023-29429-8 (PMC9902457; doi:10.1038/s41598-023-29429-8)
Supplement: Supplementary file 1 — Supplementary Information. [file 41598_2023_29429_MOESM1_ESM.pdf]

Hedonism as a Motive for Information Search — Biased Information-Seeking Leads to Biased Beliefs: Supplementary Materials

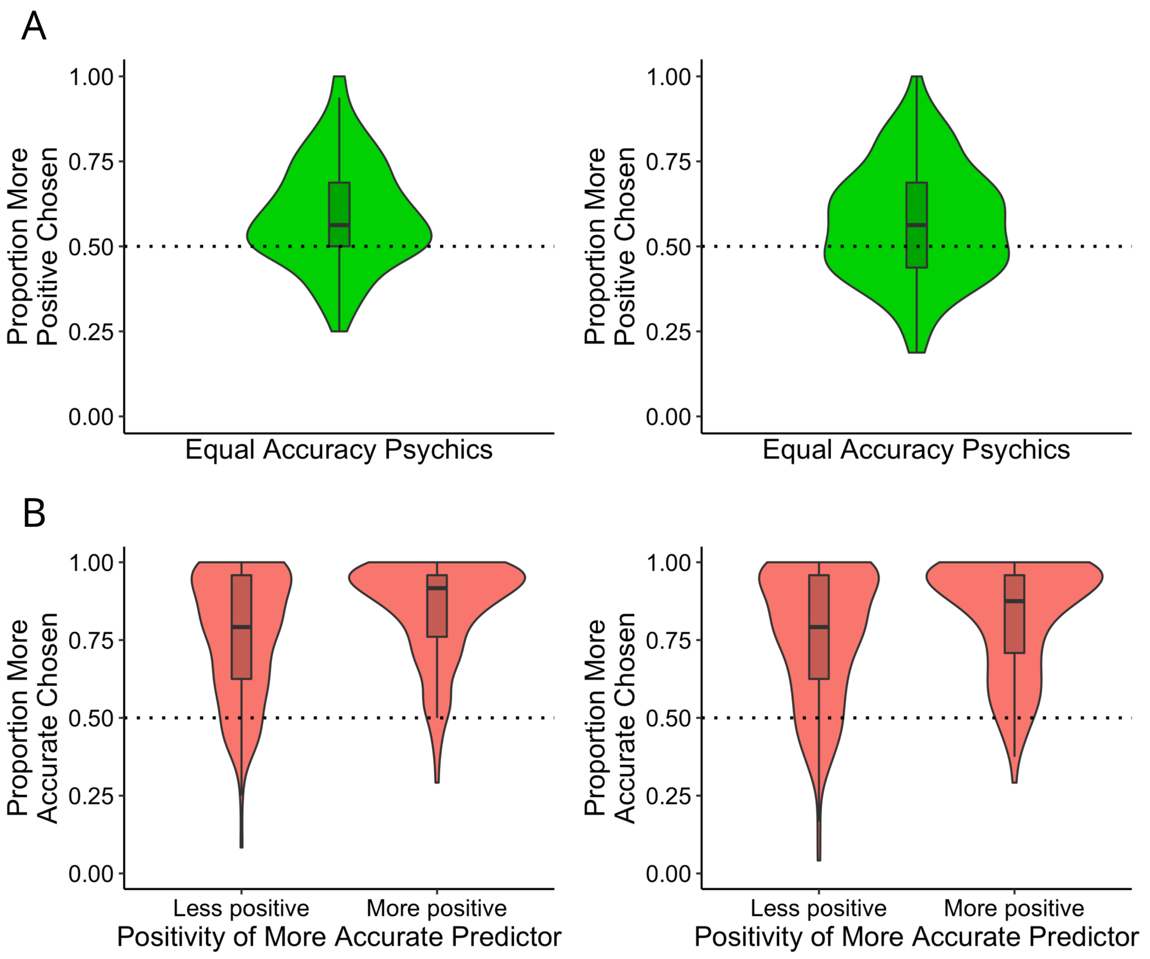

Figure A 1. **Posterior predictive check for the *Accuracy & Positivity Model.*** Plots in the left column display observed data, plots in the right column display model predictions. A) Trials in which the two psychics had equal accuracy in their example predictions. B) Trials in which the two psychics were not equally accurate, split by whether the more accurate psychic predicted fewer or more "win" outcomes than the less accurate psychic.

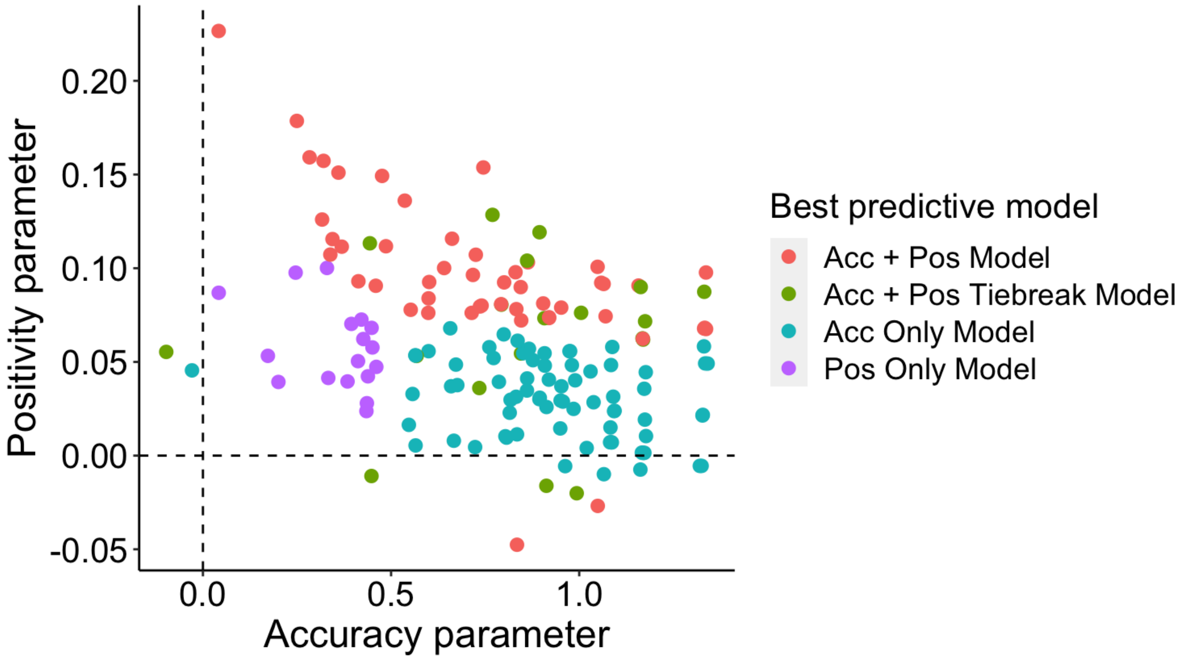

Figure A 2. **Final model parameters from the *Accuracy & Positivity Model.*** Colours correspond to which model provided the most accurate predictions of each participant's data.

Table A 1

Model recovery analysis – proportion of participants best fit by each model. *For model recovery analyses, 50 datasets were generated according to each of the four models tested. To ensure model comparison was robust to the inclusion of multiple different generative models, all four datasets were combined into a single dataset of 200 for model fitting. Model fitting procedures were the same as those used in the primary analyses. Results indicated a strong ability of the model comparison procedure to correctly identify the true generative model, with a mean accuracy of 91.5%.*

		**Best-Fitting Model**			
		Accuracy Only	Positivity Only	Accuracy & Positivity	Accuracy & Positivity Tiebreak
**Generative Model**	Accuracy Only	.86	-	.04	.10
	Positivity Only	-	.96	.02	.02
	Accuracy & Positivity	.02	-	.92	.06
	Accuracy & Positivity Tiebreak	.08	-	-	.92

Table A 2

Model recovery analysis – estimated expected log posterior density by generative and predictive model. *Results indicated that each generative model was successfully identified by the model comparison procedure.*

		Expected Log Posterior Density			
		Accuracy Only	Positivity Only	Accuracy & Positivity	Accuracy & Positivity Tiebreak
**Generative Model**	Accuracy Only	-1567	-2247	-1597	-1621
	Positivity Only	-2229	-2093	-2128	-2219
	Accuracy & Positivity	-1547	-2156	-1448	-1557
	Accuracy & Positivity Tiebreak	-1709	-2219	-1701	-1574

Table A 3

Model recovery analysis – Bayesian stacking weights by generative and predictive model. *Results indicated that each generative model formed a large majority (average of 92.95%) of the optimal weighted model when predicting the data it generated.*

		Bayesian Stacking Weights			
		Accuracy Only	Positivity Only	Accuracy & Positivity	Accuracy & Positivity Tiebreak
**Generative Model**	Accuracy Only	.854	-	-	.146
	Positivity Only	-	1	-	-
	Accuracy & Positivity	.038	-	.962	-
	Accuracy & Positivity Tiebreak	-	-	.098	.902

Table A 4

Parameter recovery analysis. *A sample of 200 participants was generated using the best fitting model (Accuracy and Positivity Model). Displayed is the rank-order correlation between the 'true' generative value and the estimated value. These values can be interpreted as reliability coefficients for parameter estimation. All parameters were reliably recovered (all $p < .001$).*

	**Parameter**		
	β	k_{Acc}	k_{Pos}
**Estimation Reliability**	.76	.71	.50

Table A 5

Combinations of trial characteristics selected for use in Experiment 1. Rows highlighted in green indicate trials in which the less accurate psychic predicts a larger number of wins than the more accurate psychic. Rows highlighted in red indicate trials in which the less accurate psychic also predicted fewer winning outcomes than the more accurate psychic. Rows highlighted in yellow indicate trials in which both psychics were equally accurate (but differed on the number of wins they predicted.

**True wins**	**Psychic 1 accuracy**	**Psychic 1 predicted wins**	**Psychic 2 accuracy**	**Psychic 2 predicted wins**
1	3	3	4	0
1	3	1	4	2
1	3	1	3	3
2	3	4	4	1
2	3	2	4	1
2	3	2	4	3
2	3	0	4	3
2	3	0	3	4
3	3	5	4	2
3	3	3	4	2
3	3	3	4	4
3	3	1	4	4
3	3	1	3	5
4	3	4	4	3
4	3	2	4	5
4	3	2	3	4

Table A 6
Combinations of trial characteristics used in Experiment 2.

True wins	Psychic accuracy	Predicted wins
2	3	0
2	3	2
2	3	4
2	4	1
2	4	3
3	3	1
3	3	3
3	3	5
3	4	2
3	4	4
